# A series of rat segmental forelimb ectopic implantation models

**DOI:** 10.1038/s41598-017-01342-x

**Published:** 2017-05-09

**Authors:** Xianyu Zhou, Xusong Luo, Bowen Gao, Fei Liu, Chuan Gu, Qingxiong Yu, Qingfeng Li, Hainan Zhu

**Affiliations:** Department of Plastic and Reconstructive Surgery, Shanghai 9th People’s Hospital, Shanghai Jiaotong University School of Medicine, Shanghai, 200011 China

## Abstract

Temporary ectopic implantation has been performed in clinical practice to salvage devascularized amputated tissues for delayed replantation purpose. In this study, we established a series of segmental forelimb ectopic implantation models in rats, including forelimb, forearm, forepaw, digit, and double forelimbs, to mimic the clinical context. Time of amputated limbs harvesting in donors and ectopic implantation process in recipients were recorded. Survival time and mortalities of recipients were also recorded. Sixty days after ectopic implantation, a full-field laser perfusion imager (FLPI) was used to detect the blood flow of amputated limbs and micro-CT imaging was used to examine bone morphological changes. Histological sections of amputated limbs were stained with hematoxylin and eosin to evaluate pathological changes. Implanted amputated limbs in all models achieved long term survival and there were no obvious morphological and histological changes were found according to results of micro-CT and histology study. Thus, a series of rat segmental forelimb temporary ectopic implantation models have been well established. To our knowledge, this is the first rodent animal model related to forelimb temporary ectopic implantation. These models might facilitate further research related to salvage, reconstruction and better aesthetic and functional outcome of upper extremity/digit in temporary ectopic implantation scenario.

## Introduction

Temporary ectopic implantation was first reported by Godina^1^ in 1986. It could be termed by temporarily implanting undamaged and devascularized amputated tissue to a secondary site of the same body with microsurgical technique to reperfuse and salvage it for later replantation to its primary anatomical site, thus achieving both aesthetic and functional results. It can be a choice to salvage the distal amputated tissue when the proximal amputated stump has suffered from severe, complicated composite tissue damages in trauma while the distal amputated part is left undamaged and devascularized^1, 2^. Temporary ectopic implantation of upper extremity^1, 3^, lower extremity^4^ and digits^5–10^ has been reported in clinical practice. This technique also has been extended the salvage and reconstruction of scalp avulsion^11, 12^, penis^13^ and free flap^14^ in plastic and reconstructive surgery. Although temporary ectopic implantation approach has been proved to be reliable and effective, animal models are still necessary to explore strategies to improve better outcome of devascularized amputated tissue when performing temporary ectopic implantation, like approaches to evaluate viability and bone morphological changes of distal amputated limb, ways to decrease implantation time and toxicity caused by ischemia-reperfusion. Murine models are commonly used in researches because of their affordability and well-established genetic backgrounds. In clinical practice, upper extremity/digits temporary ectopic implantation is the most commonly performed surgical procedure. Thus, here we presented a series of rat segmental forelimb temporary ectopic implantation models. The main goals were included: (1) Investigation of vascular anatomy and operation time related to segmental forelimb temporary ectopic implantation. (2) Observation of macroscopic features of implanted limbs during the first 60 days. (3) Evaluation of microscopic features of implanted limbs with laser perfusion, microangiography and histological examination.

## Results

### Vascular anatomy study related to segmental forelimb ectopic implantation

For forelimb ectopic implantation, the average diameter and length of the axillary artery were approximately 1 mm and 1 cm, respectively; measurements for the axillary vein were about 1.3 mm and 1 cm. The axillary vessels could be easily identified between the upper arm muscles and pectoralis major; its main branches are the lateral thoracic, thoracoacromial, and thoracicdorsal vessels. The anatomical locations and diameters of the axillary vessels were consistent. They could also be used as a vascular pedicle for the forelimb, forearm, forepaw and digit ectopic implantation.

The main vessels supplying the forearm part are branches from the axillary vessels extending to the elbow, as well as the branches of the brachial vessel including deep brachial vessels, some small branches to the elbow joint, and cutaneous branches to the overlying skin. The anatomical location of the pedicle was superficial and consistent. The pedicle was approximately 3 cm in length, making it easier for anastomosis use.

The major vessels supplying the forepaw were branches from the axillary vessels and brachial vessels extending to the forepaw. The main branches from the brachial vessel in the forearm region were the median and ulnar vessels, whose subsequent branches included the interosseous vessels responsible for partial blood supply for elbow joint, and other branches which supply forelimb muscles. The average length of the pedicle was about 5 cm. The ulnar vessel runs between the ulna and superficial head of flexor digitorum profundus, then accompanies the ulnar nerve to the manus. The median vessel runs along the medium nerve and between the flexor carpi radialis and the superficial head of flexor digitorum profundus. Both vessels became superficial at the distal part of the forearm.

The saphenous vessels had three main branches at the inferior margin of the adductor muscles. The most superior of these runs anterior to the tibia and supplies all digits, while the other two branching vessels run medially along the forearm and extend branches to the muscle, joints, and skin. Both run along the tibia nerve and curve posteriorly to the malleolus medialis, then run along the medial side of the plantar surface, where they merge into the medial plantar vessel. The medial plantar vessel divides into four branches at the midpoint of metatarsals, the first of which supplied blood to hallux. The number and location of the vessels that supplied the hallux was consistent.

The external jugular vein and the axillary vein merged at the margin of the first rib, which is a very challenging location to isolate and separate the vessels. After entering the chest wall, they are called the left and right vena cava superior, respectively. The ascending aorta, aortic arch, descending aorta, and main branches from the aorta (including the left subclavian, left common carotid, and the brachiocephalic arteries) were easily identified. All vessels within the chest wall had a consistent anatomic location and could be designed as pedicles for the double-forelimb ectopic implantation.

### Surgical time of harvesting amputated limbs in donors and implanting them in recipients

**Figure 1:**
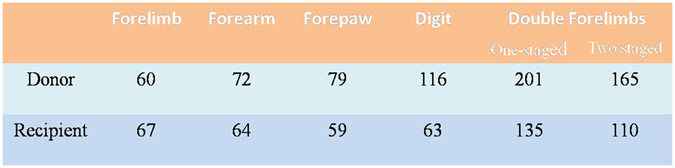
Average operation time (min) for harvesting amputated limbs in donors and implanting them in recipients in different types of temporary ectopic implantation models.

The average operation time to harvest amputated limbs in donors was about 60, 72, 79, 116, 201, 165 minutes for the forelimb, forearm, forepaw, digit, one-staged double forelimbs and two-staged double forelimbs transplants, respectively. In the recipients, the operation time of implanting the amputated limbs was about 67, 64, 59, 63, 135 and 110 minutes for forelimb, forearm, forepaw, digit, one-staged double forelimbs and two-staged double forelimbs ectopic implantation, respectively (Fig. 1).

### Macroscopic features of implanted limbs

**Figure 2:**
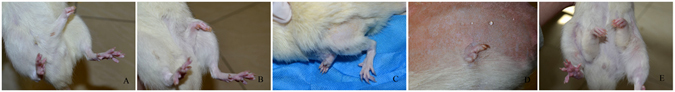
Amputated limbs from different ectopic implantation models sustained viable after operation. (**A**) Forelimb ectopic implantation (**B**) forearm ectopic implantation (**C**) forepaw ectopic implantation (**D**) first digit ectopic implantation (**E**) double forelimbs ectopic implantation.

**Figure 3:**
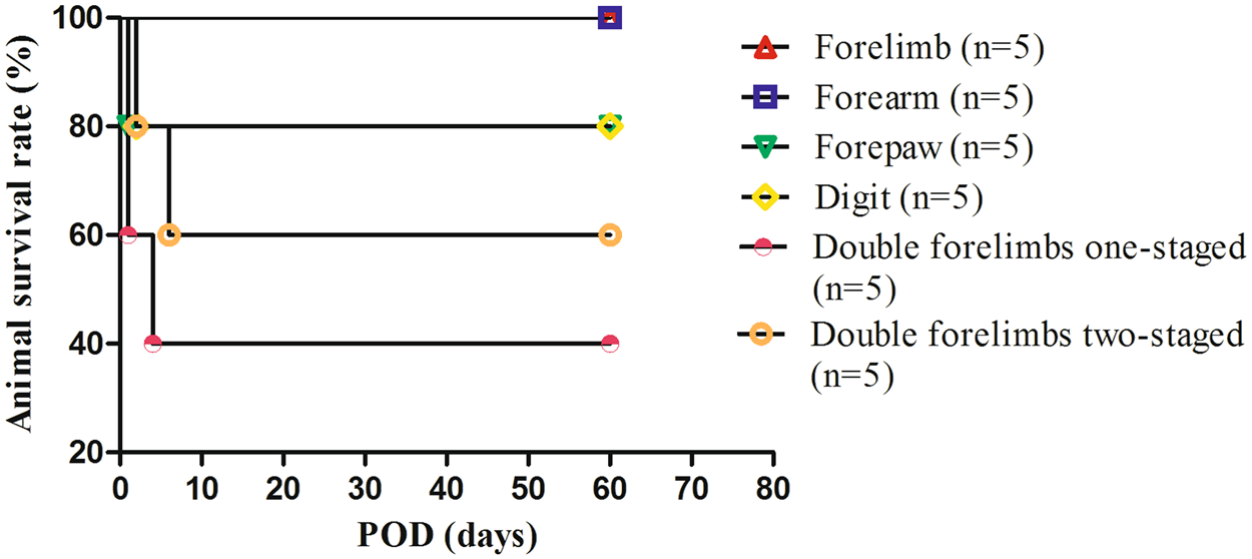
Animal survival rates in different types of ectopic implantation models.

Amputated limbs from donors in each model achieved long term survival after ectopic implantation (Fig. 2). Edema was observed in the first 3 to 5 days after operation in all models. All amputated limbs were pink and pliable during the entire observation period. Nail and hair growth was observed within 20 to 25 days after ectopic implantation. No joint rigidity was seen at the end of the experiment. All amputated limbs in the forelimb and forearm ectopic implantation groups achieved long term survival. One amputated limb was lost in the forepaw and first digit ectopic implantation groups at POD 1 and POD 2, respectively. In the double forelimbs ectopic implantation group, two recipient rats in the one-staged surgical mode did not recover from the surgery and one was lost at POD 4, while two recipient rats were lost in the two-staged mode at POD 2 and POD 6, respectively. The animal survival rate in each type ectopic implantation group was showed as follows (Fig. 3).

### Microscopic features of the implanted limbs

#### *Full-field laser perfusion imager* (*FLPI*)

**Figure 4:**
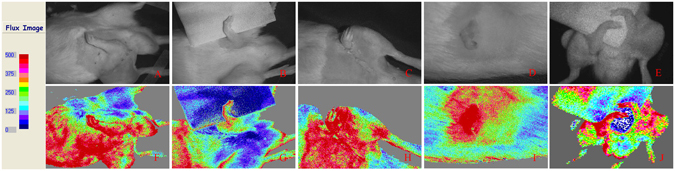
BPU of FLPI showed good blood supply to amputated limbs of different ectopic implantation models in rats. (**A**) Forelimb ectopic implantation (**B**) forearm ectopic implantation (**C**) forepaw ectopic implantation (**D**) first digit ectopic implantation (**E**) double forelimbs ectopic implantation.

The contrast images were processed to produce a color-coded live flux image (Fig. 4; the red color represented high flow speed and perfusion, and blue denoted low flow speed and perfusion). All amputated limbs denoted red color, thus representing high flow speed and perfusion with high BPU value of more than 400.

#### Microangiography of amputated limbs

**Figure 5:**
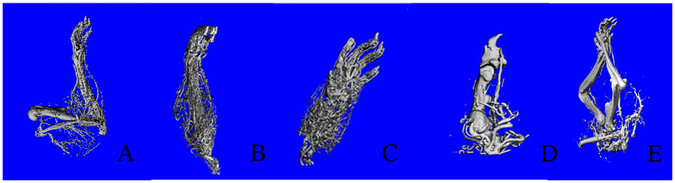
Microangiography of amputated limbs on POD60 in recipients. (**A**) Forelimb ectopic implantation (**B**) forearm ectopic implantation (**C**) forepaw ectopic implantation (**D**) first digit ectopic implantation (**E**) double forelimbs ectopic implantation.

Micro-CT scanning of amputated limbsbefore the operation and on POD 60 revealed similar morphologies. Angiographic evaluations after Microfil perfusion demonstrated good blood supply of amputated limbs (Fig. 5).

#### Histological examination

**Figure 6:**
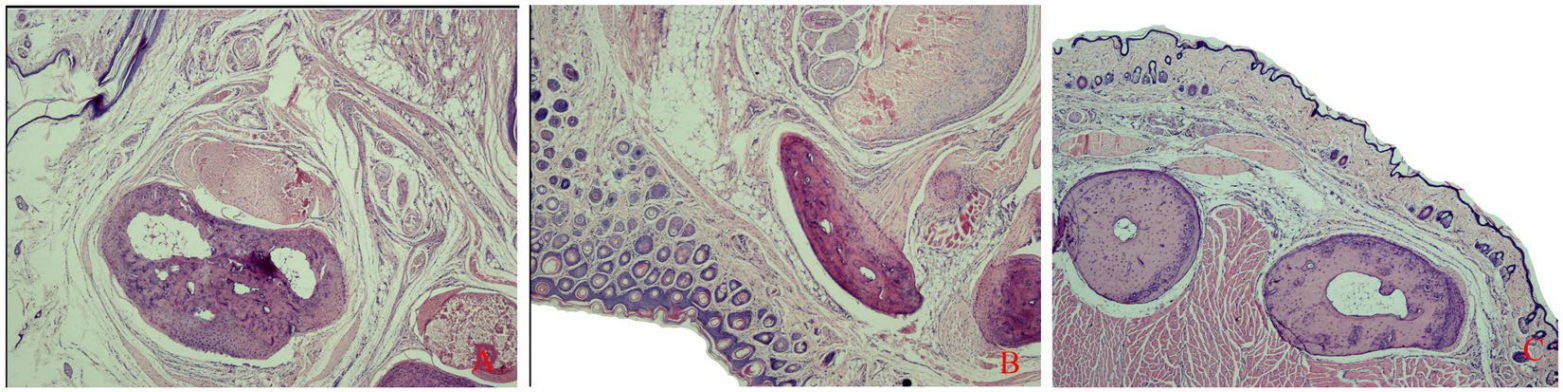
H&E staining of biopsies from different segmental sections of ectopic implantation forelimbs. (**A**) Biopsy was harvested from the forelimb. (**B**) Biopsy was harvested from the forearm. (**C**) Biopsy was harvested from the waist.

Histological specimens revealed viable bone without signs of ischemic necrosis on POD 60. Skin, subcutaneous connective tissue, vessels, tendons, and muscles showed normal structure without signs of necrosis or any other pathological changes, indicating that the amputated limbs were well vascularized (Fig. 6).

## Discussion

Godina^1^ reported the first successful temporary ectopic implantation case, in which the thoraco-dorsal vessel in the axilla was selected for the ectopic implantation of an amputated hand. However, axilla is not a good implantation site. It is always preferable for the implantation site to stay away from the movement of joint and amputation stump, otherwise it will lead to less stable circumstances, thus increasing the risks of haematomas formation or failure of the microsurgical anastomosis.

### Implantation site

Chernofsky^2^ chose the inferior epigastric vessels and Narushima^8^ chose the superficial circumflex iliac artery perforator (SCIP) and deep inferior epigastric artery perforator (DIEP) in groin as the implantation vessels. Radial vessel in the forearm is an ideal choice for the ectopic implantation as its proper vascular diameter, long pedicle and the great flexibility of reaching to amputated stump or reconstructive primary site^5, 9^. Theoretically, the blood vessel for implantation should be easily accessible and be of similar diameter with the vessels of amputated limb^8^. In our study, femoral vessel was chosen as the implantation vessel for different types of ectopic implantation models as it fulfilled all the requirements that necessary to be a good implantation vessel (except one-staged double forelimbs ectopic implantation with side-to-end anastomosis fashion).

### Temporary ectopic implantation or vascularized composite allotransplantation (VCA)

Ectopic implantation is needed when distal amputated limb is in good condition but the proximal amputated stump is in such a bad condition that needs to be reconstructed firstly. On the contrary, if the distal amputated limb was severely damaged but the proximal amputated stump or wound bed remained good condition, it is impossible to reconstruct with ectopic implantation as the distal amputated limb is lost or unreachable. To face and upper extremity with devastating composite tissue damage, the best choice may be the vascularized composite allotransplantation (VCA), which allows for complete replacement of specialized structures with “like-with-like ” but not amenable to traditional techniques^15, 16^. Another significant difference between temporary ectopic implantation and VCA is that VCA requires the involvement of immunosuppressants to prevent the rejection after allogeneic transplantation. Severe damage to different parts of the body results in completely different reconstructive approaches.

### Temporary ectopic implantation time

The time of ectopic implantation is very crucial and the minimum 2 months was recommended for the complete healing in the implantation site and sufficient revascularization for amputation part to avoid complications that may occur in a shorter implantation time^1^. In clinical practice, the implantation time is at least 4 weeks, depending on implantation site, implantation vessel and general condition of amputated limb as well as the patient himself. In our study, all the amputated limbs were implanted in groin area for 8 weeks, which conformed to the recommended time. There were no obvious morphological differences before and after ectopic implantation for the amputated limbs, indicating that there was no obvious bone degeneration, joint dislocation or fracture in a short period of time after ectopic implantation (2 months) without nerve coaptation.

### Forelimb ectopic implantation rat model

In a narrow sense, the conception of ectopic implantation restricts amputated limb to be implanted to the same body in a non-anatomical site. In our study, the amputated limb was harvested from the donor rat and implanted to recipient (another rat) other than the donor itself as it is considered as a violation of animal welfare to take the forelimb parts off and implant to a non-anatomical site of its body. Forelimb loss or severe damage to the upper extremity will lead rodent animal’s life largely impaired. The donor and recipient rats used in this study coming from the same inbred strain which is genetically identical and fundamentally free of genetic variations. The restriction of animal welfare and inbred background rat strain entitles the models we established here are actually ectopic implantation models; even though the surgical procedures could also be regarded to be syngeneic and heterotopic. According to *Greene’s Anatomy of the Rat*, forelimbs between human and rat are very similar in terms of musculoskeletal system, innervations and circulatory system^17^; as such, we also believed the rat is an excellent animal model for studying upper extremity ectopic implantation.

### Different animal mortalities

In double forelimbs ectopic implantation models, one-staged operation (two forelimbs were harvested as a whole piece) seems more difficult than two-staged operation (two forelimbs were harvested and implanted separately and sequentially); it did avoid additional vascular anastomosis but prolonged operation time because of the complicated process of separating vascular pedicle and side-to-end anatomosis pattern. Overall, the double forelimbs ectopic implantation groups experienced the highest mortality when compared with other groups (see Fig. 3); this was likely due to the increased blood loss, blood stolen phenomenon, and longer ischemia-reperfusion time.

To our knowledge, this is the first segmental rat forelimb ectopic implantation models ever reported. Salvage of amputated limb is necessary because not only do surgeons want to reconstruct the primary amputated site with amputated limb itself but also reconstitute motor and sensory function of amputated tissue with the lowest cost. As actually there is no nerve coaptation for temporary ectopic implantation, even though most of the time nerves are usually kept in amputated limb, it is really necessary to combine ectopic implantation procedure with delayed replantation procedure to assess the final functional recovery of peripheral nerves regeneration in this scenario in future study based on the model Kern *et al*.^18^ established recently. This may serve as valuable experimental tools for investigating temporary ectopic implantation of upper extremity both for animal research and clinical practice in the future.

## Conclusion

In this study, a series of rat segmental forelimb temporary ectopic implantation models have been successfully established. These models involve different segmental parts of upper extremity including digit, which may replicate the clinical forelimb ectopic implantation scenarios performed at multiple levels of the arm, and may also facilitate further experimental studies related to upper extremity.

## Methods

### Animals and anesthesia

Inbred male Lewis rats (weighting 250 to 275 g) expressing the RT1^I^ gene were obtained from the Sino-British SIPPR/BK Lab. Animal Ltd, Shanghai, China, and were allowed free access to food and water *ad libitum* in a 12-hour-light, 12-hour-dark cycled room. Animals used in this study received care in compliance with the *Guide for the Care and Use of Laboratory Animals* published by the National Institutes of Health. Isofluorane inhalation (3% for induction; 2% for maintenance) was used for general anesthesia. The experiment was approved by the Institutional Animal Care and Use Committee of Shanghai Jiaotong University School of Medicine. Donor rats were euthanized with an overdose of 5% isofluorane inhalation followed by cervical dislocation after amputated tissue harvesting.

### Experimental design

Recipient Lewis rats were randomly divided into five groups, which included forelimb, forearm, forepaw, digit, and double forelimb ectopic implantation, respectively. Five recipients were included in each of the first four groups, and ten rats were included in the double forelimbs ectopic implantation group. Of these, five received two forelimbs which were harvested as one piece in a one-staged operation mode; the other five received two forelimbs which were harvested as two pieces of forelimbs sequentially and implanted in a two-staged operation mode. Five types of ectopic implantation models were performed according to relevant anatomy study. Recipient rats received a subcutaneous injection of meloxicam (2 mg/kg) before and after the operation to relieve pain and inflammation.

### Vascular anatomy studies

**Figure 7:**
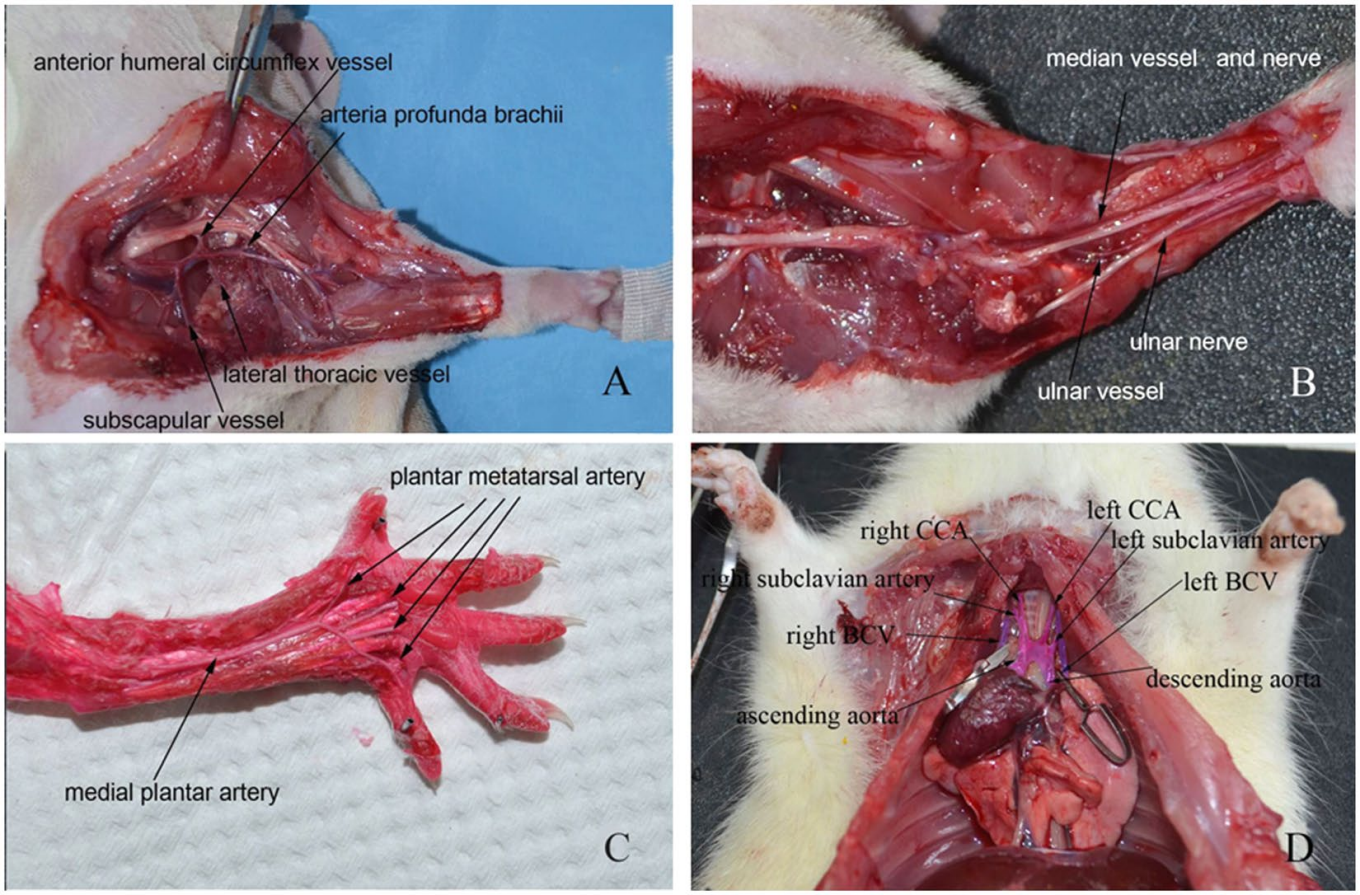
Vascular anatomy study of rat forelimb. (**A**) At the plane of axillary segment, the axillary vessel branches into the anterior humeral circumflex vessel, arteria profunda brachii, lateral thoracic vessel, and subscapular vessel. (**B**) At the plane of elbow segment, the ulnar nerve, ulnar vessel, median vessel, and median nerve were exposed. (**C**) At the plane of wrist segment, the medial plantar artery and plantar metatarsal arteries were clearly noted. (**D**) The aortic arch includes the ascending aorta and descending aorta, and branched into the CCA and subclavian artery. CCA: common carotid artery. BCV: brachiocephalic vein.

Red latex vascular perfusion was used to study the perfusion of naive rat upper extremities. The vascular pedicles of amputated forelimb, forearm, forepaw, and digit extend from the axillary to brachial, ulnar, and median vessels. These pedicles were elevated and dissected in donor rats at their entrance into the chest wall. In vascular studies of whole forelimbs, the common carotid artery and external jugular vein were identified and recorded. The vascular distribution of the femoral vessels, which acted as the recipient vessels in five types of ectopic implantation, was also identified and recorded (Fig. 7).

### Harvesting of amputated limbs in donors

Median, ulnar and radial nerves were dissected at middle point of humerus and preserved in forelimb, forearm, forepaw and double forelimbs parts while median and radial nerves in first digit part (nerve branches innervated other digits were removed) when harvesting. All amputated limbs were flushed with cold heparinized lactated Ringer’s solution (50 U heparin in 10 ml of lactated Ringer’s solution) through the artery until the outflow from vein became clear. The amputated limbs were then stored at 4 °C for 60 min to 120 min until ectopic implantation.

#### Amputated forelimb

**Figure 8:**
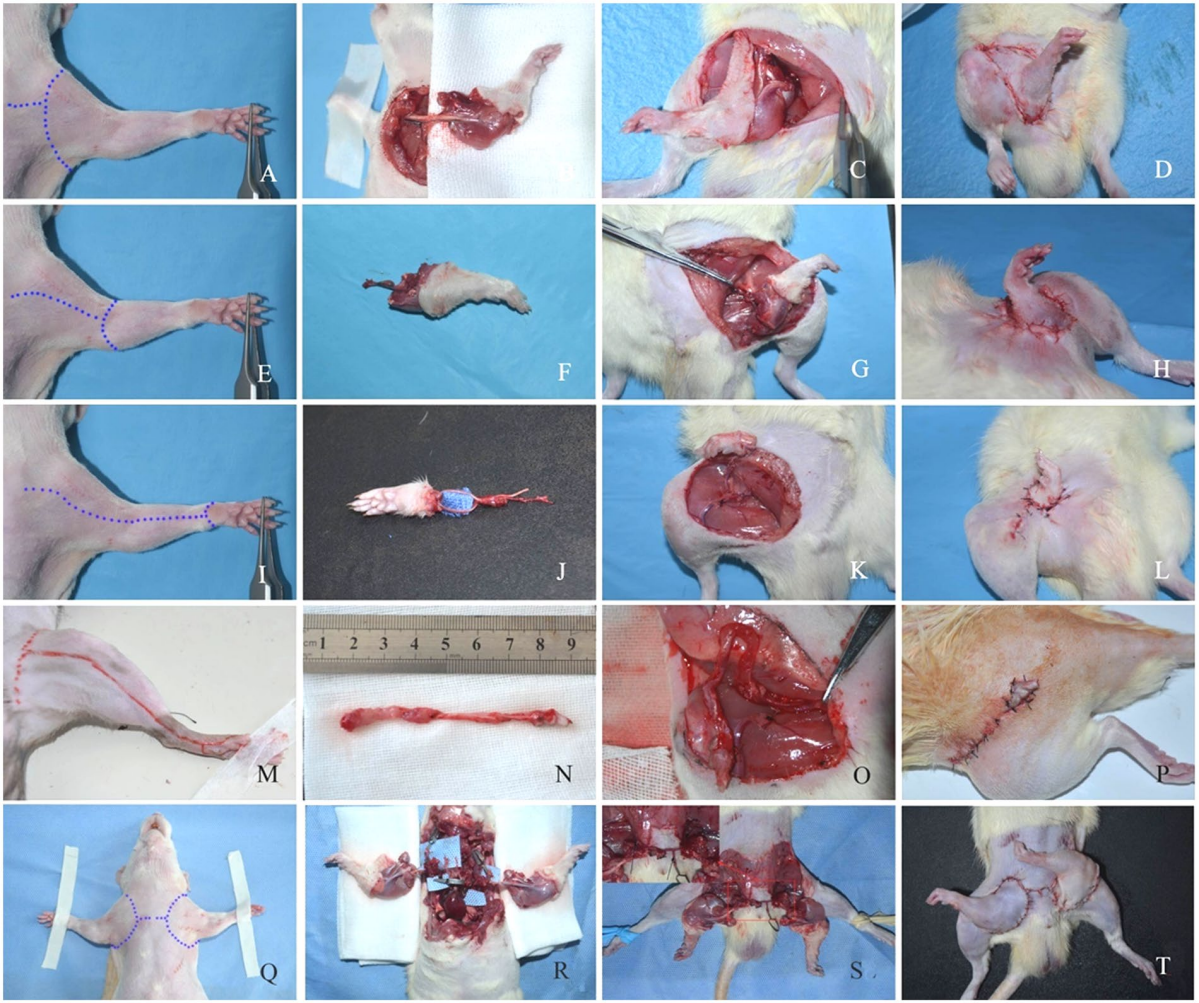
Schematic operative procedures of different ectopic implantation models. The forelimb ectopic implantation model (**A**–**D**), forearm ectopic implantation model (**E**–**H**), forepaw ectopic implantation model (**I**–**L**), first digit ectopic implantation model (**M**–**P**), double forelimbs (one-staged) ectopic implantation model (**Q**–**T**).

Incisions were made in forelimb donor rats as seen in Fig. 8(A–D.) These include a circumferential incision around the shoulder joint, as well as a short incision connecting this to the chest wall. The axillary vessel was identified below the pectoralis major and isolated from its origin at the chest wall near the shoulder joint. The lateral thoracic vessels, subscapular vessels, and thoracoacromial vessels were isolated and cauterized. The shoulder joint was then disarticulated with arm tendons intact for forelimb harvesting.

#### Amputated forearm

Incisions were made in forearm donor rats as seen in Fig. 8(E–H.) These include a circumferential incision around the elbow joint and another incision in the medial side of the arm from the elbow joint to the chest wall. The axillary and brachial vessels were isolated from their origin and traced to the elbow joint. The lateral thoracic, subscapular, and thoracoacromial vessels, as well as muscular branches from the brachial and deep brachial vessels were isolated and cauterized. The extensor and flexor muscles were divided at their origins, followed by disarticulation of the elbow joint for forearm harvesting.

#### Amputated forepaw

Incisions were made in forepaw part donor rats as seen in Fig. 8(I–L). These include a circular incision around the wrist, and another incision on the medial side of the forelimb from the wrist to the chest wall. The axillary and brachial vessels were isolated from their origins and traced to the elbow joint as described in the amputated forearm harvesting procedure. Extensor and flexor muscles were detached from their origins, and flexor muscles were removed until the median and ulnar vessels were exposed in the forelimb region. Next, these two vessels were carefully separated to the wrist plane. Other muscles of the forelimb were also dissected and removed, and the radial and ulnar bones were cut off distally with a rotary saw.

#### Amputated first digit

The incisions shown in Fig. 8(M–P) were made from the axillary region to the medial wrist in donors. Then the incision was curved posterior to the medial malleolus and continued to the midpoint of the first digit of the upper extremity. The dissection was performed from dorsum to plantar sequentially. The first dorsal metatarsal artery was cauterized, the extensor hallucis longus divided, and then the first metatarsophalangeal joint was disarticulated. The first metatarsal bone was removed to expose the muscular layers under this bone. The lumbrical muscles, adductor hallucis, flexor hallucis brevis, and the flexor hallucis longus tendon were identified and removed. Then the medial plantar vessels, the first metatarsal artery and vein were exposed, branches from the medial plantar vessels to the other digits were cauterized. Finally, the plantar aponeurosis and plantar skin were dissected, and then whole first digit was amputated and harvested.

#### Amputated double forelimbs

Double-forelimb ectopic implantation was performed in either one- or two-staged operations. In one-staged operation, left and right forelimbs were harvested as one piece of graft (Fig. 8Q–T). The axillary vessels of both forelimbs were isolated to their origins at the entrance of the chest wall. The chest wall of the donor rat was opened with caution, and the thymus, adipose tissue, lymph nodes over heart and large vessels were removed. The ascending aorta, aorta arch, descending aorta, brachiocephalic trunk, bilateral common carotid arteries, bilateral subclavian arteries, superior vena cava, and bilateral brachiocephalic veins were exposed and isolated. The descending aorta, bilateral common carotid artery, and the external jugular vein were dissected and ligated, while the ascending aorta and bilateral brachiocephalic veins were prepared as donor vascular pedicles. Due to the thin wall of the brachiocephalic vein and its intense adhesion to the pleura visceralis, 2 to 3 ribs were cut to keep the donor vessels intact. In two-staged operation, the harvesting process of each amputated forelimb was the same as described in the “amputated forelimb” section.

### Bed preparation and amputated limbs insetting in recipient

Femoral vessels were exposed in recipient rats through an incision in either the right or left inguinal region. Recipient and donor vessels were anastomosed in an end-to-end pattern using 11–0 microsurgical sutures (S&T, 03195) under a surgical optical microscope. When the ascending aorta was used as pedicle for one-staged double forelimbs ectopic implantation, a wedge anastomosis fashion (end-to-side pattern) was performed due to the obvious mismatch of vessel sizes between donor and recipient. Amputated limbs were placed in the inguinal region with the least tension for forelimb, forearm, forepaw and double forelimbs ectopic implantation. Amputated digit was placed on the back of the recipient rat in the forepaw ectopic implantation model. Nerves in amputated tissues of each model were kept without coaptation. Skin wounds were closed using 6–0 non-absorbable sutures (Ethicon).

### Survival evaluation of amputated limbs

All amputated limbs were monitored daily by gross observation and blood flow using capillary refill technique to assess for viability after ectopic implantation. The general health and weight of the animals were also monitored every three days.

### Full-field laser perfusion imager (FLPI) features of amputated limbs

A full-field laser perfusion imager (FLPI, Moor instrument, UK) utilizes a 780 nm laser beam to measure the average blood speed in tissue and the concentration of traveling red blood cells in a tissue sample volume by performing contrast analysis on images acquired from a Charge-coupled Device (CCD) video camera. All measurements were performed in a warm (24 °C) and quiet environment. The CCD camera was positioned 30 cm above the tissue surface of each rat; settings for low-resolution/high-speed images included a display rate of 25 Hz, time constant of 1.0 s and camera exposure time of 20 ms. The height and system parameters remained constant in every case with values expressed in BPU. The contrast images were processed to produce a color-coded live flux image (red denoted high perfusion, blue signified low perfusion). The measurements were performed on POD 30.

### Microangiography features of amputated limbs

On POD 60, amputated limbs were harvested and scanned with a Scanco micro-CT 40 (SCANCO Medical AG, Zurich, Switzerland) at 20-μm resolution with a segmentation threshold of 312. Spatial segmentation of the contrast within the amputated tissue was assessed, and the three-dimensional bone structure was reconstructed. Amputated limbs of arm were subsequently perfused with Microfil. The general circulation was flushed and replaced using consecutive intracardiac injection of heparinized saline, saline and 10% formaldehyde, respectively. Microangiography was performed by infusing a synthetic endovascular polymer (Microfil 1, Flow Tech, Carver MA). After polymerization for 24 h at 4 °C, the amputated limbs were again scanned and reconstructed using micro-CT for gross examination of the vascular pattern.

### Histological evaluation

After Microfil angiography on POD 60, tissue specimens were harvested from all recipients for histological evaluation. Samples of the composite tissue components of forelimb were harvested, fixed in 10% formalin solution for 3 days, and then decalcified in Immunocal solution (Decal Chemical Corporation, Tallman, NY) for 14 days. Fixed specimens from mid-forelimb, wrist, and forepaw were then embedded in paraffin and cut into 7-μm cross-sections. Sections were then stained with hematoxylin-eosin and evaluated for signs of ischemia or any other pathological changes.
